# Preventable hospitalizations, barriers to care, and disability

**DOI:** 10.1097/MD.0000000000010691

**Published:** 2018-05-11

**Authors:** Liliana E. Pezzin, Hillary R. Bogner, Jibby E. Kurichi, Pui L. Kwong, Joel E. Streim, Dawei Xie, Ling Na, Sean Hennessy

**Affiliations:** a Department of Medicine and Center for Patient Care and Outcomes Research (PCOR), Medical College of Wisconsin, Milwaukee, WI; b Department of Family and Medicine & Community Health, Perelman School of Medicine, University of Pennsylvania, PA; c Center for Clinical Epidemiology and Biostatistics, Perelman School of Medicine; d Center for Pharmacoepidemiology Research and Training; e Geriatric Psychiatry Section of the Department of Psychiatry, Perelman School of Medicine, University of Pennsylvania, Philadelphia, PA.

**Keywords:** ambulatory-care sensitive conditions, barriers to care, disability, preventable hospitalizations

## Abstract

The AHRQ's Prevention Quality Indicators assume inpatient hospitalizations for certain conditions, referred as ambulatory-care sensitive (ACS) conditions, are potentially preventable and may indicate reduced access to and a lower quality of ambulatory care. Using a cohort drawn from the Medicare Current Beneficiary Survey (MCBS) linked to Medicare claims, we examined the extent to which barriers to healthcare are associated with ACS hospitalizations and related costs, and whether these associations differ by beneficiaries’ disability status. Our results indicate that the regression-adjusted cost of ACS hospitalizations for elderly Medicare beneficiaries with no disabilities was $799. This cost increased six-fold, by $5148, among beneficiaries with mild disability, by $9045 for beneficiaries with moderate disability, by $5513 for those with severe disability, and by $8557 for persons with complete disability (*P* < 0.001). Persons reporting having foregone or delayed needed medical care because of financial difficulties (+$2082, *P* = .05), those experiencing low satisfaction with care coordination (+$1714, *P* = .01), and those reporting low satisfaction with access to care (+$1237, *P* = .02) also incurred significant excess ACS hospitalization costs relative to persons reporting no such barriers. This pattern held true for those with and without a disability, but were especially marked among persons with no functional limitations. These findings suggest that a better understanding of how public policy might effectively improve care coordination and reduce financial barriers to care is essential to formulating programs that reduce excess hospitalizations among the large and growing number of elderly Medicare beneficiaries.

## 1 Introduction

The Prevention Quality Indicators, established by the Agency for Healthcare Quality and Research (AHRQ) in 2011, assume that inpatient hospitalizations for certain conditions, referred to as ambulatory-care sensitive (ACS) conditions, are potentially preventable and may indicate reduced access to and a lower quality of ambulatory care. ACS conditions are defined as those “for which good outpatient care can potentially prevent the need for hospitalization, or for which early intervention can prevent complications or more severe disease."^[1–3]^ Endorsed by the Institute of Medicine,^[4]^ lists of these conditions have been used as indicators in the United States, Canada, England, Brazil, and other countries.^[5–10]^

Hospitalizations for ACS conditions have been applied to track potentially preventable hospitalizations in a variety of vulnerable populations,^[11–16]^ but not among those with disability. Evidence suggests that people with disabilities are more likely to perceive accessibility barriers and have poorer health status.^[17,18]^ They are also more likely to have lower incomes, and less social and emotional support, factors that may further contribute to experiencing barriers to timely, coordinated, high-quality ambulatory care services.^[19,20]^ Such barriers to care may manifest as increases in potentially preventable hospitalizations for complications typically manageable in outpatient settings. Because excess hospitalizations have substantial quality of life, health, and economic implications to the society at large, reduction of ACS hospitalizations is both a quality metric and a cost-saving policy priority.

The purpose of this study was to determine the extent to which barriers to healthcare are associated with ACS hospitalizations and related costs, and whether these associations differ by beneficiaries’ disability status. We hypothesize that barriers to care will independently and positively affect the probability and overall cost of ACS hospitalizations. We further anticipate that this relationship will be greater among persons at higher levels of disability.

## 2 Methods

### 2.1 Data source and study population

Data for this study were drawn from the Medicare Current Beneficiary Survey (MCBS), a rotating panel, longitudinal, nationally representative survey of Medicare beneficiaries conducted by the Centers for Medicare and Medicaid Services (CMS).^[21]^ Sampled persons are interviewed 3 times a year over the course of 4 years following their entry into the survey. Survey data, available from MCBS’ Access to Care files, are subsequently linked to Medicare claims data compiled under MCBS's Cost and Use files, which provide comprehensive information on each beneficiary's healthcare service utilization and expenditure data. For each survey respondent, inpatient, outpatient, and noninstitutional claims data are available for 3 consecutive years, starting on the first calendar day of the year following the initial survey wave, which occurs in the Fall.

We focus on entry cohorts of community-dwelling beneficiaries aged 65 years or older from the 2001 to 2007 waves of the MCBS, for whom complete information on 3-year healthcare utilization was available from MCBS's records. The sample was weighted to be representative of the fee-for-service elderly Medicare beneficiaries who were living in the community. The study was approved by the appropriate Institutional Review Boards.

### 2.2 Outcome measures

Our primary outcomes were any ACS hospitalization and overall costs associated with ACS hospitalizations. For both, information was obtained from MCBS’ Cost and Use files which include hospitalizations derived from a combination of survey reported healthcare utilization and Medicare administrative files. Applying the most recent set of Prevention Quality Indicators established by the Agency for Healthcare Research and Quality (AHRQ),^[22]^ we defined ACS hospitalizations as those secondary to complications of existing chronic ACS conditions (i.e., diabetes short-term complications, diabetes long-term complications, uncontrolled diabetes, lower extremity amputation secondary to diabetes, chronic obstructive pulmonary disease (COPD)/asthma, hypertension, congestive heart failure, and angina without procedures) as well as those resulting from an acute ACS condition (dehydration, bacterial pneumonia, and urinary tract infection). For each identified ACS hospitalization, we used the associated Cost and Use file event claim's total payments (rather than charges) as our measure of cost, regardless of the source of payment (Medicare only, Medicaid (dual enrollees), supplemental, or other insurance). ACS hospitalizations and costs were assessed for all individuals over a 3-year period following the beneficiary's entry into the survey. To account for changes over time, annual costs were adjusted using the Medical Care Component of the Consumer Price Index standardized to the end-point of the study period.^[23]^ Inflation-adjusted person-level costs were then aggregated over all ACS hospitalizations that occurred during the study period, thereby reflecting resource utilization associated with overall length of stay across (potentially multiple) ACS hospitalizations.

### 2.3 Barriers to care

We measure self-reported barriers to healthcare in 5 dimensions: care coordination and quality, access to care, financial considerations, transportation difficulties, and usual source of care. The first 2 dimensions are summary scores derived from beneficiaries’ responses to 8 MCBS questions about perceived quality and access barriers, each with scores ranging from 1 to 4 for very satisfied to very unsatisfied. The care coordination and quality score combines the average self-reported responses to 5 questions about overall quality of care, information given, follow-up care, concern for overall health, and needs met at the same location. The access to care score is formed by 3 questions including the availability of night/weekend, ease/convenience, and out-of-pocket costs. Both summary scores were derived from factor analyses applied to MCBS surveys using a principal components method with oblique (Promax) rotation to allow for correlation among the dimensions.^[24]^ Following similar work,^[25,26]^ we classify beneficiaries with an average summated score corresponding to the upper quartile as being completely satisfied, and use this group as reference category when contrasting the experience of beneficiaries in the 3 lower quartiles who were classified as less satisfied and, therefore, experiencing some barrier with respect to a particular dimension of care.

Beneficiaries indicating that they either had trouble getting healthcare in the previous year because of cost or had delayed needed medical care in the past year because of cost were classified as experiencing a financial barrier to healthcare. Beneficiaries who indicated that transportation to the doctor's office or hospital was a reason for trouble getting needed healthcare in the previous year were coded as experiencing transportation barriers. Finally, beneficiaries indicating that they did not have a particular place where they “received medical care, such as a doctor's office, group practice, or doctor's clinic” were coded as having no usual source of care.

### 2.4 Other covariates

Variables capturing sociodemographic and economic characteristics include age, gender, race/ethnicity, and an indicator of poverty status as measured by dual (Medicare and Medicaid) enrollment. The number of comorbidities was based on self-reported information about the presence of the following medical conditions: Alzheimer's/dementia, angina/chronic heart disease, diabetes mellitus, emphysema/asthma/COPD, hypertension, mental/psychiatric disorder, mental retardation, myocardial infarction, nonrheumatoid arthritis, osteoporosis/soft bones, other heart disease, cancer of a site other than skin, Parkinson's disease, rheumatoid arthritis, and stroke.

Using the algorithm develped by Stineman et al^[27]^ we classified all beneficiaries, hierarchically, into 5 activity limitation stages based on the nature and severity of their ADL disability. Classification into each activity limitation stage was based on the respondent's answers about the level of difficulty experienced when performing the 6 ADL activities: eating, toileting, dressing, bathing/showering, getting in and out of bed/chairs, and walking. Higher activity limitation stages reflect greater disability and less preserved function. Specifically, persons in stage 0 have no limitation, those in stage I have mild limitation, stage II moderate limitation, and stage IV complete limitation. Respondents who did not follow the typical hierarchy of loss of function were assigned to a nonfitting stage, stage III. Details about the derivation of the activity limitation stages can be found elsewhere.^[27,28]^ All covariates were measured at the entry survey.

### 2.5 Statistical analysis

We relied on Duan's two-part model^[29,30]^ to examine the independent effect of barriers to care and disability on ACS hospitalizations and associated costs, controlling the array of patient's sociodemographic, economic, and health characteristics defined above. The first part models the probability of any ACS hospitalization during the study period, while the second part models (conditional) ACS hospitalization costs among beneficiaries who had at least one ACS hospitalization during the study period. The two-part model is an attractive alternative specification to traditional ordinary least squares and Tobit regressions as it addresses the econometric challenges posed by ACS hospitalization cost data including its restricted range (non-negative observations), the relatively large proportion of zero values, and the skewness caused by a small number of very high cost cases. Following Buntin and Zaslavsky,^[31]^ we use a Probit specification to model the probability of any ACS hospitalization (first part) and a generalized linear model for inferences about factors associated with conditional costs.^[32,33]^

To test our hypothesis that the relationship between barriers to care and cost of ACS hospitalizations differ by the beneficiaries’ disability status, in a second set of two-part models, we included an indicator variable capturing disability (activity limitation stages I–IV relative to stage 0), barriers to care, and an interaction term between these variables (representing the differential effect of each given barrier on persons with or without activity limitations).

Based on parameter estimates from these two-part models, we then computed *unconditional* (adjusted) marginal effects of barriers to care, activity limitation stages, and interactions between disability status and barriers to care on 3-year ACS hosptialization costs.^[34]^ All analyses used weighted data and were performed using SAS 12.4 and STATA 14 statistical software (College Station, TX).

## 3 Results

### 3.1 Sample characteristics

A total of 22,248 respondents, corresponding to about 41.5 million Medicare beneficiaries over the age of 65 years old, participated in the 2001 to 2007 waves of the MCBS and were included in the study. Of those, 2460 (11.1%), corresponding to slightly over 4.6 million elderly beneficiaries) had a preventable ACS hospitalization during the 3-year period following their survey entry. Table 1 shows the summary characteristics of the sample overall and by ACS hospitalization.

**Table 1:**
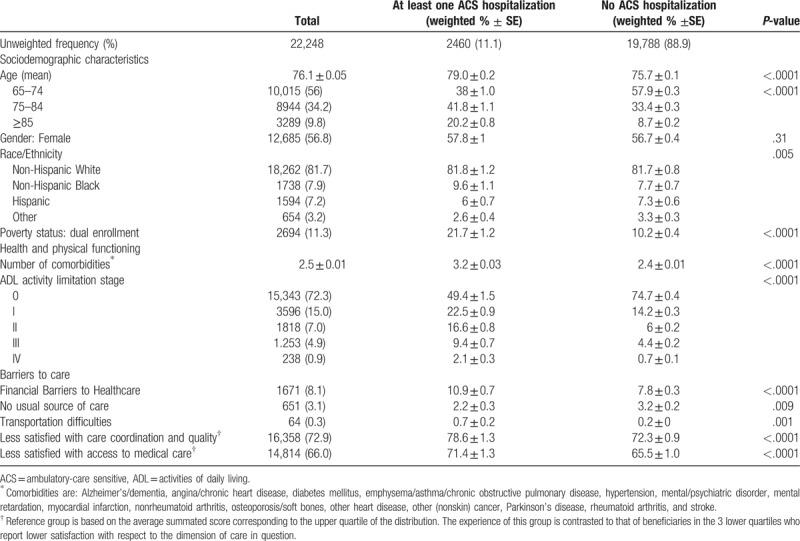
Summary statistics, overall and by ACS hospitalization, among 2001–2007 MCBS respondents aged 65 or older at survey entry.

Among these cohorts of elderly Medicare beneficiaries, over a half were aged 65–74 years old and nearly 10% were 85 or older. The vast majority (81.7%) were white, most were women (56.8%) and had no ADL disability as captured by activity limitation stage 0 (72.3%). About one in 10 beneficiaries was enrolled in both Medicare and Medicaid. Despite public insurance coverage, 8.1% (or 3.4 million Medicare beneficiaries over the 2001–2007 study period) reported having trouble getting or having delayed seeking needed medical care because of financial difficulties, and 3.1% reported having no usual source of care. By virtue of dichotomizing the within-sample scores by upper (completely satisfied) and lower quartiles, 72.9% of the weighted sample was not completely satisfied with the care coordination and quality of medical services received and 66% was not completely satisfied with access to care. (These numbers were not exactly 75% because of ties.) Less than 1% reported having a transportation barrier that kept them from receiving healthcare.

Summary statistics by ACS hospitalization reveal differences across groups of beneficiaries experiencing (vs not) a preventable hospitalization by age groups, race/ethnicity, poverty, and health status, including the number of comorbidities and activity limitation stage. Most notably, compared to beneficiaries reporting no barriers to healthcare, those who experienced financial barriers were significantly more likely to experience at least one ACS hospitalization within the 3-year study period following their survey entry (10.9% vs 7.8%, respectively). Similarly, relative to beneficiaries without each specific barrier, those reporting transportation difficulties (0.7% vs 0.2%) as well as those who reported being less than completely satisfied with care coordination and quality (78.6% vs 72.3%) and access to care (71.4 vs 65.5%) were significantly more likely to experience at least one ACS hospitalization within the study period (Table 1).

### 3.2 Factors associated with probability and costs of ACS hospitalizations

Table 2 presents coefficient estimates for each of the 2 parts of the Duan two-part model: probability of at least one ACS hospitalization and (conditional) overall cost of ACS hospitalizations among beneficiaries who had one or more ACS hospitalizations during the 3-year study period. (We opted for presenting results of the probability of ACS hospitalization based on a logistic regression results due to ease of interpretation. Substantive findings were identical between the logit and the probit specification used in the two-part model.)

**Table 2:**
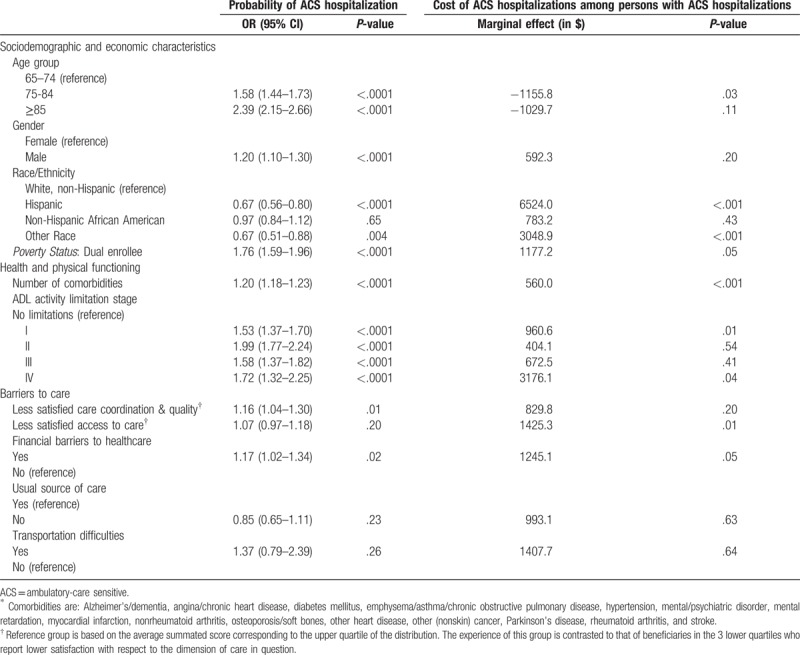
Factors associated with probability and cost of ACS hospitalizations.

Elderly Medicare beneficiaries who were less satisfied with care coordination and quality were more likely than those completely satisfied to have at least one ACS hospitalization within the study period (odds ratio (OR)=1.16; 95% confidence interval (CI): 1.04–1.30). Similarly, persons who reported having delayed or not sought needed medical care because of financial difficulties were statistically significantly more likely to have an ACS hospitalization than those who did not experience such financial barriers (OR = 1.17; 95% CI:1.02–1.34). Other statistically significant factors associated with increased likelihood of an ACS hospitalization include older age (85+ years old, OR = 2.39; 95% CI:2.15–2.66), dual enrollment (OR = 1.76; 95% CI:1.59–1.96), disability status (OR = 1.53, 1.99, 1.58 and 1.72, for mild (stage I), moderate (stage II), nonfitting (stage III) and complete (stage IV) activity limitation stage, respectively), male gender (OR = 1.20; 95% CI: 1.10–1.30), and higher number of comorbidities (Table 2, column 1). Relative to their non-Hispanic white counterparts, elderly beneficiaries of Hispanic descent as well as those of other race/ethnicities were less likely to have at least one ACS hospitalization in the 3-year period.

Among the subset of 2460 beneficiaries with at least one ACS hospitalization, persons reporting lower satisfaction with access to care incurred ACS hospitalization costs that were $1425 (*P = *.01) higher than those completely satisfied with their access to care. Similarly, persons who reported financial considerations as reason for delaying or not seeking needed medical care had (adjusted) ACS hospitalization costs that were, on average, $1245 (*P = *.05) higher than those of beneficiaries reporting no financial barriers to healthcare. Although the point estimates of the conditional cost of ACS hospitalizations were higher for all elderly beneficiaries with activity limitations, the coefficients were statistically significant only for those with mild (stage I, +$961, *P = *.01) or severe (stage IV, +$3176, *P = *.04) disability relative to those with no activity limitations in ADLs (stage 0).

Despite a lower likelihood of having an ACS hospitalization, the cost of ACS hospitalizations among Hispanic beneficiaries and those of other races/ethnicities was statistically significantly higher than of non-Hispanic white beneficiaries ($6524 and $3049, *P < *.001, respectively). Conditional on having at least one such inpatient stay, the cost of ACS hospitalizations was $1177 (*P = *.05) higher among dual enrollees. Finally, once comorbidities, activity limitations, poverty status, and barriers to care were controlled for, older age was generally associated with lower conditional costs of ACS hospitalizations.

### 3.3 Barriers to care, disability, and overall cost of ACS hospitalizations

To put our results into perspective, we computed (predicted) marginal effects of disability and barriers to care on overall unconditional cost of ACS hospitalizations for elderly Medicare beneficiaries based on coefficients from the two-part model described above. Specifically, for each activity limitation stage and barrier to care, we calculated individual-level predicted probabilities of an ACS hospitalization from the first part model, holding other factors constant at their original values. A similar approach was used to obtain marginal effects of each of these variables on expected, conditional ACS hospitalization costs from the second part of the model. Predicted unconditional marginal costs were calculated by multiplying these 2 components (Table 3, upper panels). In addition, to quantify the contribution of alternative barriers to care by the beneficiary's disability status, we also computed unconditional cost of ACS hospitalizations based on two-part models that included interaction terms between certain key barriers to care (i.e., those significantly associated with either the probability of at least one or the conditional cost of ACS hospitalizations: care coordination and quality, access to care, financial barriers) and disability status (stages I–IV relative to stage 0), controlling for all the other factors in the original model. Results of these calculations are shown in the lower panel of Table 3.

**Table 3:**
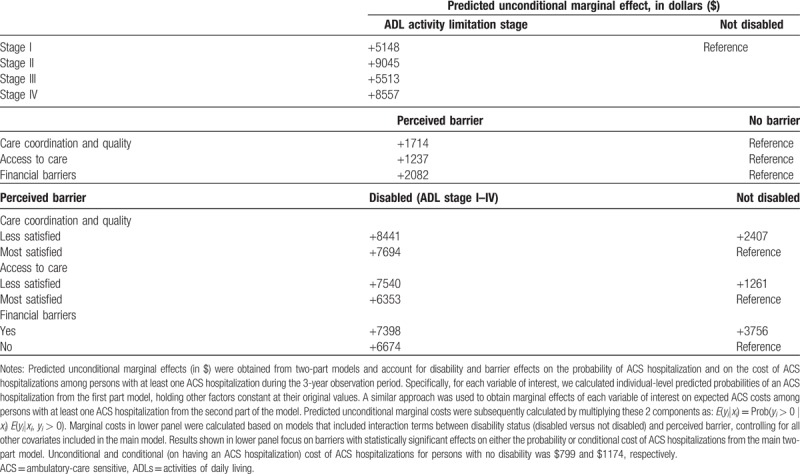
Unconditional marginal effects of disability status and barriers to care on cost of ACS hospitalizations.

The predicted (unconditional) cost of ACS hospitalizations for elderly Medicare beneficiaries with no disability (stage 0 ADL activity limitation) was $799. This cost increased sixfold, by $5148, among beneficiaries with mild disability (Stage I ADL activity limitation), by $9045 for beneficiaries with moderate (Stage II) disability, by $8557 for persons with complete disability (Stage IV), and by $5513 for those in the nonfitting Stage III disability. These differences represent the independent effect of activity limitation stage on 3-year ACS hospitalization costs, after controlling for barriers to care, comorbidities, and other confounding variables described above.

Cost differentials were also marked for barriers to care. Persons reporting having foregone or delayed needed medical care because of financial difficulties incurred excess ACS hospitalization costs of $2082 relative to those reporting no such financial barriers. The independent, marginal effect of experiencing lower satisfaction with care coordination and quality and access to care on excess ACS hospitalization costs was $1714 and $1237, respectively, relative to beneficiaries reporting being completely satisfied with each dimension of healthcare.

Relative to elderly beneficiaries with no ADL disabilities who were most satisfied with the coordination and quality of the care their received, being less satisfied with care coordination and quality lead to an excess ACS hospitalization cost of $2407. This value corresponds to the net excess cost of experiencing a barrier with care coordination and quality among persons with no disability, holding all other factors constant at their original levels. Despite their substantially higher excess ACS hospitalization costs (+$8441 and +$7694), the excess ACS hospitalization cost of experiencing the same barrier among beneficiaries with ADL limitations was about one-third of that of persons without such activity limitations ($8441-$7694 = $747).

Excess costs of ACS hospitalizations were the largest among persons with no ADL limitations who experienced financial barriers to care. On average, ACS hospitalizations for this group cost $3756 more than those of persons with no disability and no financial barriers. Here again, although having a disability increased substantially the cost of ACS hospitalizations among those with or without financial barriers (+7398 and +$6674, respectively), the excess cost of delaying or foregoing needed care because of financial difficulties was relatively small ($724) when compared to the effect of the same barrier on persons without a disability.

## 4 Discussion

Understanding the relationship between barriers to timely and affordable healthcare and ACS hospitalizations among elderly persons is an important social and policy concern, particularly in view of recent discussions about further cuts in the Medicare program. Although there is a growing literature on identifying ambulatory-care sensitive conditions, their prevalence and associated costs, it has largely focused on quantifying the extent of racial, ethnic, geographic, and socioeconomic disparities therein based on hospital discharge or state-specific data.^[11–16,35,36]^ Evidence suggests that ACS hospitalization rates are highest among African–American, Hispanic, and other minority populations. Excess preventable hospitalizations are also correlated with residence in rural areas as well as in areas characterized by low primary care physician density, large minority populations, and high poverty levels. With few notable exceptions,^[37,38]^ little attention has been paid to disability status as a source of disparities in ACS hospitalizations, and no US study has examined potentially preventable hospitalizations as a result of barriers to healthcare.

In this study, we used a nationally representative sample of elderly Medicare beneficiaries to fill in this gap. Our findings reveal that persons at higher stages of disability face substantially higher odds of experiencing an ACS hospitalization than those with no activity limitations. However, among those with at least one preventable hospitalization, the severity of the disability was not consistently associated with higher (conditional) costs. Persons reporting barriers with care coordination and quality, barriers with access to timely ambulatory care, as well as those reporting having foregone or delayed medical care because of financial difficulties incurred substantially higher overall ACS hospitalization costs than those without such barriers, a pattern that held true for those with and without a disability. The most striking finding, however, was the sizeable association between lower satisfaction with care coordination and quality and financial barriers with overall (unconditional) ACS hospitalization costs among persons *without* activity limitations.

Appropriate management of ACS hospitalizations depends on timely and effective organization of healthcare. One implication of our findings is that efforts to improve care coordination and expand access to subsidized care among the general population of elderly Medicare beneficiaries is likely to yield substantial cost savings by promoting a more efficient use of health care resources. In fact, based on marginal effects applied to the number of elderly Medicare beneficiaries with no activity limitations in 2015, we estimated that about US $58.8 million could be saved over a period of 3 years in ACS hospitalizations if persons experiencing barriers with care coordination and quality had the same probability and conditional cost of preventable ACS hospitalizations as those with no such barriers (46.3 million elderly beneficiaries ∗ 0.723 without limitations ∗ 0.73 with care coordination barriers ∗ $2407 unconditional marginal effect among persons without limitations). Similarly, the resulting cost savings from fewer or shorter ACS hospitalizations had elderly Medicare beneficiaries with a financial barrier had the same utilization as those without such a barrier would be approximately US $10.2 millions.

Our study differs from previous investigations in important ways. Rather than relying on counts of ADLs and IADLs, we used a newly developed and validated activity limitation staging system that is based on clinical thresholds and accounts for the nature and type of disability to classify elderly Medicare beneficiaries according to disability stage.^[27]^ In addition, our sample comprises several recent cohorts of MCBS respondents, from which we documented disability stage and self-reported barriers to care, linked to Medicare claims, from which we ascertained ACS hospitalizations and associated costs. Several limitations merit comment, however. Our study evaluated ACS hospitalizations and related costs among a sample of elderly Medicare beneficiaries receiving care in the fee-for-service environment. Because HMO and Medicare Advantage plans are not required to submit claims to CMS, these beneficiaries were excluded from the study. In addition, disability stage was assessed by self-reported information on difficulty in performing certain activities of daily living, which may be subject to measurement error. Finally, we did not examine temporal trends in ACS hospitalizations. To the extent that such hospitalizations may have changed over time during the study period, our results reflect the average, rather than the marginal, probability of ACS hospitalizations among beneficiaries at different stages of disability facing different barriers to care.

## 5 Conclusion

Considered potentially avoidable, ACS hospitalizations have been increasingly used to indicate low quality of preventive care. Our analysis suggests that enhanced care coordination and greater access to affordable care have the potential to reduce preventable hospitalizations among elderly Medicare beneficiaries. A better understanding of how public policy might effectively reduce such barriers is essential to formulating programs that meet the goal of providing affordable, timely access to services for the large and growing number of elderly Medicare beneficiaries.

## Author contributions

Liliana E. Pezzin designed the study and conducted the econometric analyses; Ling Na, Dawei Xie, and Pui Kwong were responsible for data acquisition, variable construction and preparing analytical files; Sean Hennessy, Hillary Bogner, Joel Streim and Jibby Kurichi provided insights into the interpretation of results and contributed important intellectual content to the final paper.

**Conceptualization:** Liliana Pezzin, Jibby E. Kurichi, Joel E. Streim, Sean Hennessy.

**Formal analysis:** Liliana Pezzin, Hillary R. Bogner, Jibby E. Kurichi, Pui L. Kwong, Joel E. Streim, Dawei Xie, Ling Na, Sean Hennessy.

**Methodology:** Liliana Pezzin.

**Writing – original draft:** Liliana Pezzin.

**Writing – review & editing:** Liliana Pezzin, Hillary R. Bogner, Jibby E. Kurichi, Joel E. Streim, Sean Hennessy.

**Validation:** Pui L. Kwong, Dawei Xie, Ling Na.
